# Effect of silver diamine fluoride and potassium iodide on shear bond strength for glass ionomer cement to primary dentine

**DOI:** 10.6026/973206300200808

**Published:** 2024-07-31

**Authors:** Snigdha Biswas, Ashwini Koti, Harish Kumar, Santoshkumar S. Kotnoor, Arvind Jain, Kritika Jaiswal, Pratik Surana

**Affiliations:** 1 Department of Periodontology and Implantology, BBD College of Dental Sciences, Lucknow, Uttar Pradesh, India; 2 Department of Pediatric and Preventive Dentistry, SMBT Dental College and Hospital, Sangamner, Maharashtra, India; 3 Department of Oral Pathology, Kalinga Institute of Dental Sciences, KIIT Deemed to be University, Bhubaneswar, Odisha, India; 4 Department of Oral Pathology and Microbiology, Hke' society S Nijalinagappa Dental College, Kalaburagi, Karnataka, India; 5 Department of Conservative Dentistry & Endodontics, Government College of Dentistry, Indore, Madhya Pradesh, India; 6 Department of Pedodontics and Preventive Dentistry, Rungta College of Dental Sciences & Research, Bhilai, Chhattisgarh, India; 7 Department of Pedodontics and Preventive Dentistry, Maitri College of Dentistry and Research Centre, Durg, Chhattisgarh, India

**Keywords:** dental caries, silver diamine fluoride, potassium iodide

## Abstract

Silver diamine fluoride (SDF) is a highly effective topical fluoride for halting dental caries; however, it darkens both teeth and restorations. Therefore, it is of interest to assess the shear bond strength (SBS) of glass ionomer cement (GIC) to caries-affected dentin treated with SDF alone and SDF followed by KI. Forty primary molar samples were prepared to reveal a flat dentin surface and were randomly assigned to two groups. In group A, the dentin surfaces were pre-treated with 38% SDF, while in group B, the dentin was treated first with SDF and then with KI before being restored with GIC. The SBS was measured using a universal testing machine. The results show that teeth pre-treated with both SDF and KI demonstrated significantly improved bond strength of GIC to dentin compared to SDF treatment alone.

## Background:

The contemporary approach to managing dental caries in pediatric patients emphasizes minimally invasive, tissue-preserving, cost-effective, and safe methodologies. Concurrently, efforts are on-going to develop more efficacious anti-caries agents and determine optimal restoration strategies [1]. SDF is a solution composed of ionic silver, fluoride, and ammonia that halts the progression of carious lesions and prevents subsequent caries formation. SDF facilitates a more conservative preparation of the tooth by effectively promoting the remineralization of residual decay [2]. The silver ions in SDF exhibit significant antimicrobial properties by disrupting the formation of cariogenic biofilm, specifically by interfering with bacterial synthesis of cellular polysaccharides. Comparative studies have demonstrated that SDF superior efficacy than fluoride varnish in caries prevention [3]. Despite the previously noted benefits of SDF application, its use is not without limitations. For instance, the resultant black stain from SDF may affect aesthetic outcomes. However, this stain can be mitigated by restoring the cavity with glass ionomer cement or composites [4]. Alternatively, immediately following SDF application, applying a supersaturated solution of potassium iodide followed by GIC restoration can also conceal the stain. This method leverages the reaction between SDF and potassium iodide to produce silver iodide (AgI) and tripotassium phosphate (K3PO4). The latter, being a white powder, effectively masks the black discoloration [5].

Cavitated lesions necessitate restorative procedures as they facilitate self-cleansing, thereby reducing the likelihood of secondary caries. Additionally, restorations help to seal out plaque that harbors bacteria and restore both aesthetics and functionality. The sustained release of fluoride from the restorative material in the marginal gaps (between the restoration and the tooth) is instrumental in preventing secondary caries. This characteristic of Glass Ionomer Cement makes it the preferred material for restorations, particularly in patients exhibiting high caries activity. [4,6] Shear bond strength refers to the resistance of restorative materials to forces that cause them to slide past the tooth structure. Clinically, SBS is critically important as a higher SBS indicates superior bonding of the restorative material to the tooth. [7] Therefore, it is of interest to assess and compare the shear bond strength of glass ionomer cement on the dentin of primary teeth preconditioned with SDF alone and in combination with potassium iodide.

## Materials and Method:

Material utilized in this research is mentioned in Table 1. The sample size was ascertained in accordance with the findings of a prior study using G* Power software (3.1).[8] The level of significance (α error) was established at 5% and the power of the study at 80% (0.8). Consequently, the total required sample size was determined to be 20 participants per group. In this study, 40 non-carious intact human primary molars, scheduled for extraction due to physiological mobility and orthodontic indications, were selected. Molars affected by caries, fractures, injuries, restorations, or hypoplastic/developmental anomalies were excluded. The chosen teeth underwent debridement of soft tissue debris and calculus via ultrasonic scaling, and then were preserved in a 0.02% thymol solution for one week, followed by storage in normal saline at ambient temperature. Subsequently, the teeth were embedded in self-curing acrylic blocks measuring 3 x 2 cm, ensuring the occlusal surfaces were aligned parallel to the surface of the resin block. The occlusal enamel was removed using a high-speed dental handpiece with a diamond bur and water coolant until dentin surface was exposed. The surface was further refined and smoothed using 600-grit silicon carbide paper. A 3 x 3 mm window was then precisely created with vernier calipers and nail varnish to expose only the flattened dentine area. Artificial dentine carious lesions were induced by a pH-cycling procedure, by immersing in a demineralizing solution (pH 4.4, 50 mM acetate, 2.2 mM KH_2_PO_4_, 2.2 mM CaCl_2_) for 7 days. The specimens were allocated into two groups through a simple randomization process based on the type of solution applied.

## Group A (SDF):

Silver diamine fluoride was administered to the tooth surface via a micro brush applicator tip, agitated for 1 minute, and left undisturbed for 2 minutes before being thoroughly rinsed with water for 30 seconds.

## Group B (SDF+ KI):

Following the application of SDF, a saturated potassium iodide solution was applied immediately. This application was repeated until the ensuing creamy white precipitate turned clear, after which the precipitate was extensively rinsed with water for 30 seconds.

Subsequently, all teeth specimens from both groups were restored using glass ionomer cement, shaped by cylindrical Teflon molds. Post-restoration, the teeth were placed into individual containers with distilled water and underwent thermocycling for 500 cycles between temperatures of 5 and 55°C, with a dwell time of 60 seconds. The samples were stored in distilled water for 2 days. Shear bond value analyzed by universal testing machine operating at a crosshead speed of 1 mm/min until the specimen was dislodged.

## Results:

The findings demonstrated that the average shear bond strength of group A, measuring 4.54 ± 1.31 MPa, was significantly inferior compared to group B, which displayed an average SBS of 8.11 ± 1.65 MPa. The mean difference of 3.57 MPa between the two cohorts was statistically significant, with a p-value of less than 0.05, as detailed in Table 2.

## Discussion:

Contemporary scholarly literature, encompassing randomized clinical trials and systematic reviews, demonstrates that the application of silver diamine fluoride is an effective strategy for halting carious lesions in pediatric populations. The chemical interaction between SDF and dental hydroxyapatite leads to the formation of calcium fluoride and silver phosphate, which play crucial roles in inhibiting and preventing dental caries.[9,10] Nonetheless, SDF's tendency to cause tooth staining poses aesthetic concerns that limit its broader utilization, despite its demonstrated caries-arresting efficacy. To mitigate this staining, potassium iodide has been introduced to address the issue. When combined with SDF, a saturated potassium iodide solution induces the formation of a creamy white precipitate of silver iodide crystals on the tooth surface, which effectively reduces the availability of free silver ions to form black precipitates, thus preventing discoloration. [2,11]The current investigation revealed a statistically significant decline in bonding efficacy within the SDF group in comparison to the SDF + potassium iodide group. This diminution in bond strength is likely due to the formation of silver deposits and silver oxide, leading to the coagulation of exposed denatured collagen fibrils. [4,8] Furthermore, silver particles penetrate the dentinal tubules, leading to their total or partial obstruction and thereby interfering with micromechanical retention, adhesive infiltration, and ultimately resulting in reduced hybrid layer formation. These findings are consistent as shown elsewhere [5]. Conversely, in the SDF+ potassium iodide group, a statistically significant improvement in bonding performance was noted, surpassing that observed in the SDF group. This enhancement can be explained by the reaction between potassium iodide and SDF, where the iodide stimulates the formation of a silver iodide precipitate within the demineralized dentin substrate. This precipitate may occlude the dentinal tubules, reducing fluid flow and thereby enhancing bonding performance. Silver diamine fluoride consequently demonstrates a wide range of applications in pediatric dentistry. Nevertheless, its utilization has been constrained due to a notable limitation, specifically tooth discoloration. Further research into potassium iodide (KI) or alternative agents that could mitigate this discoloration is warranted [8].

## Conclusion:

Data shows that pretreatment of demineralized primary dentin with SDF and KI affect the bonding performance of GIC positively.

## Figures and Tables

**Table 1:** Illustration of material used in study

**Name of material**	**Composition/specification**	**Company/Manufacture**
SDF	Silver 24-28% Ammonia 5-6%	e-SDF, Kids-e-Dental, Mumbai, India
KI	Potassium iodide in deionized water	Lugol's solution, India
GIC	Calcium, fluoroaluminosilicate powder and Polyacrylic acid (aqueous solution), tartaric acid, water liquid	GC Fuji II, GC Corporation, Tokyo

**Table 2:** Comparative evaluation of mean shear bond strength

**Group**	**Mean Shear bond Strength**	**Mean Difference**	**P Value**
SDF	4.54 ± 1.31 Mpa	3.57	<0.05*
SDF + KI	8.11 ± 1.65 Mpa		
*Significant
